# Cellulose Nanocrystal/Zinc Oxide Bio-Nanocomposite Activity on Planktonic and Biofilm Producing Pan Drug-Resistant *Clostridium perfringens* Isolated from Chickens and Turkeys

**DOI:** 10.3390/antibiotics14060575

**Published:** 2025-06-03

**Authors:** Ismail Amin, Adel Abdelkhalek, Azza S. El-Demerdash, Ioan Pet, Mirela Ahmadi, Norhan K. Abd El-Aziz

**Affiliations:** 1 Microbiology and Parasitology Department, Faculty of Veterinary Medicine, Badr University in Cairo (BUC), Badr City 11829, Egypt; ismail.amin@buc.edu.eg; 2 Food Safety, Hygiene and Technology Department, Faculty of Veterinary Medicine, Badr University in Cairo (BUC), Badr City 11829, Egypt; adel.abdelkhalek@buc.edu.eg; 3 Laboratory of Biotechnology, Department of Microbiology, Agricultural Research Center (ARC), Animal Health Research Institute (AHRI), Zagazig 44516, Egypt; dr.azzasalah@yahoo.com; 4 Department of Biotechnology, Faculty of Bioengineering of Animals Resources, University of Life Sciences “King Mihai I” from Timisoara, 300645 Timisoara, Romania; mirelaahmadi@usvt.ro; 5 Department of Microbiology, Faculty of Veterinary Medicine, Zagazig University, Zagazig 44511, Egypt

**Keywords:** *C. perfringens*, necrotic enteritis, pan drug-resistance, biofilm formation, green nanoparticles, CNCs/ZnO nanocomposite, real-time quantitative PCR

## Abstract

**Background/Objectives:** *Clostridium perfringens* is a normal inhabitant of the intestinal tract of poultry, and it has the potential to induce cholangiohepatitis and necrotic enteritis (NE). The poultry industry suffers significant financial losses because of NE, and treatment becomes more challenging due to resistant *C. perfringens* strains. **Methods:** The antimicrobial and antibiofilm activities of cellulose nanocrystals/zinc oxide nanocomposite (CNCs/ZnO) were assesses against pan drug-resistant (PDR) *C. perfringens* isolated from chickens and turkeys using phenotypic and molecular assays. **Results:** The overall prevalence rate of *C. perfringens* was 44.8% (43.75% in chickens and 58.33% in turkeys). Interestingly, the antimicrobial susceptibility testing of *C. perfringens* isolates revealed the alarming PDR (29.9%), extensively drug-resistant (XDR, 54.5%), and multidrug-resistant (MDR, 15.6%) isolates, with multiple antimicrobial resistance (MAR) indices ranging from 0.84 to 1. All PDR *C. perfringens* isolates could synthesize biofilms; among them, 21.7% were strong biofilm producers. The antimicrobial potentials of CNCs/ZnO against PDR *C. perfringens* isolates were evaluated by the agar well diffusion and broth microdilution techniques, and the results showed strong antimicrobial activity of the green nanocomposite with inhibition zones’ diameters of 20–40 mm and MIC value of 0.125 µg/mL. Moreover, the nanocomposite exhibited a great antibiofilm effect against the pre-existent biofilms of PDR *C. perfringens* isolates in a dose-dependent manner [MBIC50 up to 83.43 ± 1.98 for the CNCs/ZnO MBC concentration (0.25 μg/mL)]. The transcript levels of *agrB* quorum sensing gene and *pilA2* type IV pili gene responsible for biofilm formation were determined by the quantitative real time-PCR technique, pre- and post-treatment with the CNCs/ZnO nanocomposite. The expression of both genes downregulated (0.099 ± 0.012–0.454 ± 0.031 and 0.104 ± 0.006–0.403 ± 0.035, respectively) when compared to the non-treated isolates. **Conclusions:** To the best of our knowledge, this is the first report of CNCs/ZnO nanocomposite’s antimicrobial and antibiofilm activities against PDR *C. perfringens* isolated from chickens and turkeys.

## 1. Introduction

One of the most important parts of the agricultural production system is the poultry industry [1]. Even though there is always a chance that broiler chickens can contract infections, particularly those caused by microorganisms typically present in their guts, they can be reduced by improved management and proper information on infectious poultry diseases [2]. There are numerous etiological agents causing enteric diseases in chickens, one of which is *Clostridium* species [3]. *Clostridium perfringens* (*C. perfringens*) is a Gram-positive, anaerobic, spore-forming bacterium that is a natural component of the poultry gut microbiota [4]. Commonly, *C. perfringens* is present in the intestines of healthy chickens in concentrations of less than 10^2^–10^4^ CFU/g of intestinal content, as opposed to 10^7^–10^9^ CFU/g in diseased birds [5].

The presence of approximately twenty different extracellular enzymes and toxins is the basis for *C. perfringens* pathogenicity. The microorganism has been classified into seven toxinotypes, namely A to G, based on the presence of major toxin genes: *cpa* (alpha), *cpb* (beta), *etx* (epsilon), *iap* (iota), *cpe* (enterotoxin), and *netB* (necrotic enteritis B-like toxin) [6,7]. Necrotic enteritis (NE) is a bacterial disease caused by strains of *C. perfringens* [4]. The disease is brought on by an excessive rise in the number of *C. perfringens* bacteria in the gastrointestinal tract (GIT) in combination with risk factors such as exposure to mycotoxins, diets high in non-starch polysaccharide grains, and coccidia infection [8]. Both clinical and subclinical forms of NE can cause high mortality rates and growth performance failures, respectively. The estimated yearly cost of NE is over 6 billion USD, or 0.05 USD per chick [4]. Control strategies for NE involve the use of antimicrobials [9]. The prolonged and extensive use of antimicrobials in poultry over the past few years has altered the bacterial environment, eliminated susceptible strains, and promoted the persistence and dominance of antimicrobial-resistant bacteria [10,11].

Biofilms can be described as the houses and cities of the bacterial world [12]. They are aggregates of microorganisms in which extracellular polymeric substances (EPS) adhere to a surface, and/or each other’s cells are frequently embedded in a self-produced matrix [13] that undergo stages of accumulation, attachment, maturation, and dispersal [14]. Bacteria can adhere to substrates and encapsulate themselves in a matrix consisting of extracellular DNA (eDNA), proteins, and EPS, when they form biofilms [15,16]. The antibiotic sensitivity of bacterial cells in biofilms is lower than that of their planktonic counterparts [16], thus, increasing the resistance to antimicrobial drugs and reducing the effectiveness of biofilm-related infection treatment. Several investigations have revealed that biofilm-producing bacteria have a resistance potential that is more than 1000 times higher than that of planktonic ones [17,18]. Antibiotic resistance arises from the slower penetration of antimicrobial drugs due to the formation of the biofilm matrix, which in turn leads to the establishment of persistent cells, a resistant phenotype [18]. Another potential mechanism for antibiotic resistance in biofilms is the upregulation of bacterial efflux pumps [19,20].

The emergence of antibiotic-resistant bacterial strains and their transmission to humans can threaten food safety and public health. Therefore, there is an urgent need to explore natural products as alternatives to antibiotics for the poultry industry.

Copper (Cu), zinc (Zn), and selenium (Se) nanometals have strong antibacterial and antibiofilm activities, which makes them viable options for treating bacterial infections, particularly those are resistant to antibiotics. Each of these nanometals exhibits distinct processes and degrees of efficiency, and their effectiveness against a range of bacteria, including both Gram-positive and Gram-negative species, has been investigated [21,22,23,24].

The most prevalent polymer on Earth is cellulose (Cs), which is also a key part of plant cell walls. Nanocellulose, also known as nanostructured cellulose, combines the characteristics of Cs and nanomaterials [25]. Now, turning our attention to ZnO nanoparticles, which are recognized for their stability and antibacterial properties, are useful in food engineering and other nanotechnology applications [26]. By inhibiting the in vitro bacterial growth and concentrating on bacterial toxin manufacture, the dual-target therapy of new cellulose nanocrystals/zinc oxide (CNCs/ZnO) bio-nanocomposites improves bacterial treatment and lowers the emergence of antibiotic resistance [27]. To the best of our knowledge, no studies have evaluated the antimicrobial and antibiofilm activities of CNCs/ZnO nanocomposite against PDR *C. perfringens* isolated from poultry by phenotypic and molecular assays.

## 2. Results

### 2.1. Prevalence of C. Perfringens in Poultry Samples

As shown in Table 1, the overall prevalence rate of *C. perfringens* was 44.8% (77/172). In broilers, *C. perfringens* isolates were detected with a percentage of 44.4% (55/124), with a higher existence in the intestine (25/124; 20.2%), followed by the liver (15/124; 12.1%) and spleen (15/124; 12.1%). While in layers, 41.6% (15/36) were positive for *C. perfringens* with a higher existence in the spleen (6/36; 16.6%) followed by liver (5/36; 13.8%) and intestine (4/36; 11.1%). On the other hand, 7 out of 12 examined turkeys (58.3%) had *C. perfringens* isolates with a higher existence in the liver (5/12; 41.6%) followed by the intestine (2/12; 16.6%), and no presence of *C. perfringens* in spleen. Statistical analysis revealed non significant difference in the prevalence of *C. perfringens* among liver, spleen, and intestine samples isolated from broiler chickens and layers (*p* = 0.122 and 1, respectively), while there was a statistically significant difference in the prevalence of *C. perfringens* among liver, spleen, and intestine samples isolated from turkeys (*p* = 0.046). Additionally, there was no statistically significant difference in the prevalence of *C. perfringens* among total broiler, layer, and turkey samples (*p* = 0.608).

### 2.2. Antibiogram of C. perfringens Isolates

**Figure 1:**
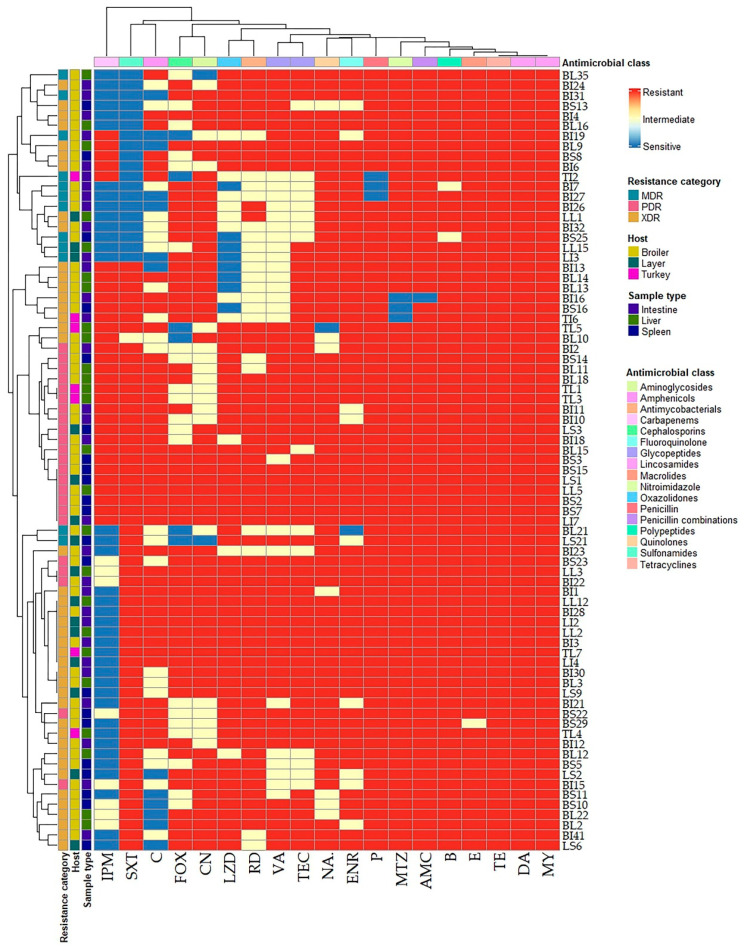
Hierarchical clustering heatmap showing the overall distribution of the investigated *C. perfringens* isolates based on the phenotypic antimicrobial resistance pattern. The code numbers on the right of the heatmap refer to the isolates from broiler liver (BL), broiler spleen (BS), broiler intestine (BI), layer liver (LL), layer spleen (LS), layer intestine (LI), turkey intestine (TI), and turkey liver (TL) samples. Different antimicrobial classes, resistance categories, hosts, and sample types are color-coded on the right of the heatmap. P: penicillin G benzylpenicillin, AMC: amoxycillin-clavulanic acid, FOX: cefoxitin, IPM: imipenem, CN: gentamicin, E: erythromycin, NA: nalidixic acid, ENR: enrofloxacin, SXT: sulfamethoxazole-trimethoprim, C: chloramphenicol, B: bacitracin, LZD: linezolid, DA: clindamycin, MY: lincomycin, TE: tetracycline, VA: vancomycin, TEC: teicoplanin, MTZ: metronidazole, RD: rifampin.

**Figure 2:**
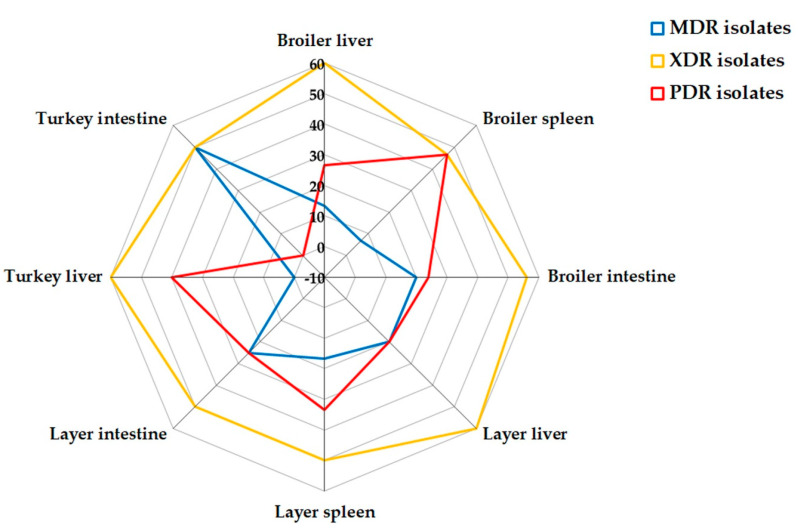
Occurrence of MDR, XDR, and PDR categories in *C. perfringens* isolated from poultry. MDR: multidrug-resistant; XDR: extensive drug-resistant; PDR: pan drug-resistant. Antimicrobial resistance categories are indicated in the colour key; green color originates from the overlapping of both blue and yellow colors, which means that green color contains both MDR and XDR isolates.

**Figure 3:**
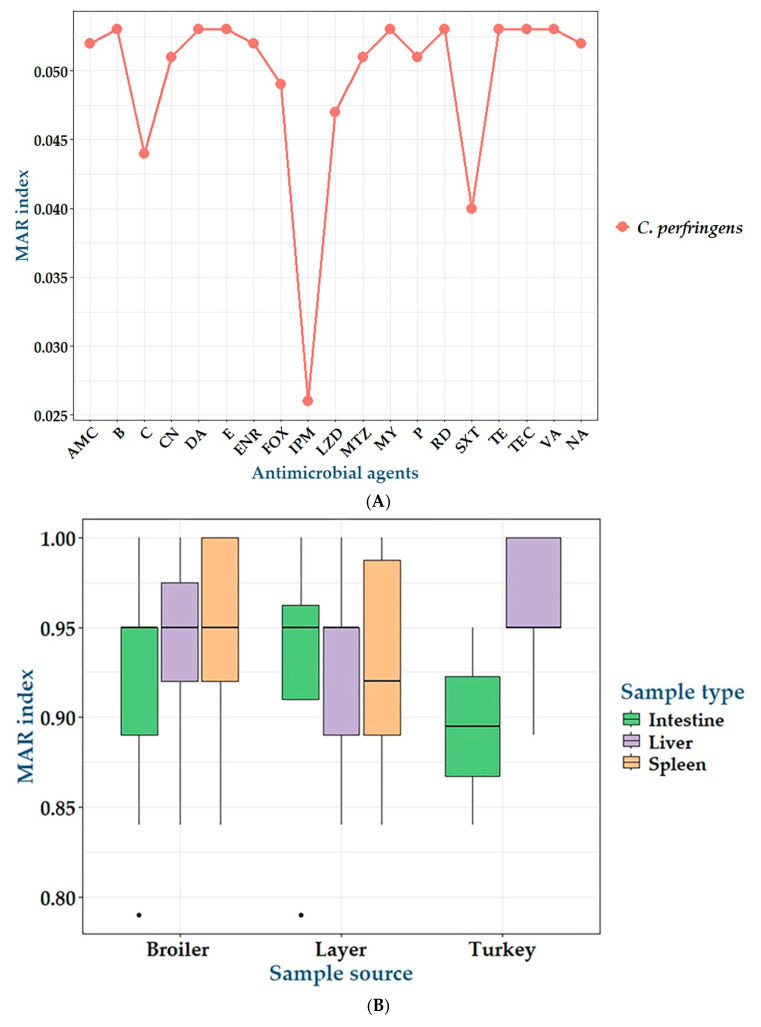
(**A**): Multiple antimicrobial resistance index (MAR index) of the tested antimicrobials against *C. perfringens* isolates. P: penicillin G benzylpenicillin, AMC: amoxycillin-clavulanic acid, FOX: cefoxitin, IPM: imipenem, CN: gentamicin, E: erythromycin, NA: nalidixic acid, ENR: enrofloxacin, SXT: sulfamethoxazole-trimethoprim, C: chloramphenicol, B: bacitracin, LZD: linezolid, DA: clindamycin, MY: lincomycin, TE: tetracycline, VA: vancomycin, TEC: teicoplanin, MTZ: metronidazole, RD: rifampin. (**B**): MAR index of the tested *C. perfringens* isolates belonging to various sources.

The in vitro antimicrobial susceptibilities of 172 *C. perfringens* isolates against 19 antimicrobial agents are summarized in Table 2 and Figure 1. The antimicrobial susceptibility testing revealed that *C. perfringens* isolates exhibited complete resistance to erythromycin, bacitracin, clindamycin, tetracycline, rifampin, vancomycin, teicoplanin, and lincomycin. Moreover, high levels of resistance were detected for amoxycillin-clavulanic acid, nalidixic acid, and ciprofloxacin (99%), gentamicin (97%), penicillin G and metronidazole (96%), cefoxitin (92%), linezolid (90%), chloramphenicol (83%), and sulfamethoxazole trimethoprim (75%). However, a moderate resistance rate was reported for imipenem (53%). Statistical analysis revealed significant variations (*p* < 0.001) in the antimicrobial susceptibilities of bacterial isolates to all tested antimicrobials except for imipenem (*p* = 0.374). Interestingly, 29.9% (23/77) of *C. perfringens* isolates were PDR, 54.5% (42/77) were XDR, while 15.6% (12/77) were MDR (Table 3 and Figure 2) exhibiting multiple antimicrobial resistance (MAR) indices ranged from 0.84 to 1 (Figure 3). There were statistically significant differences (*p* < 0.05) in the prevalence of various resistance categories among *C. perfringens* isolates from different origins. Additionally, there were no statistically significant differences in the occurrence of MAR indices (*p* > 0.05) among *C. perfringens* isolates obtained from liver, spleen, and intestine samples from broiler chickens, layers, and turkeys. Also, there were no statistically significant differences in the occurrence of MAR indices (*p* = 1 each) among *C. perfringens* isolates obtained from broiler, layer, and turkey origins.

**Figure 4:**
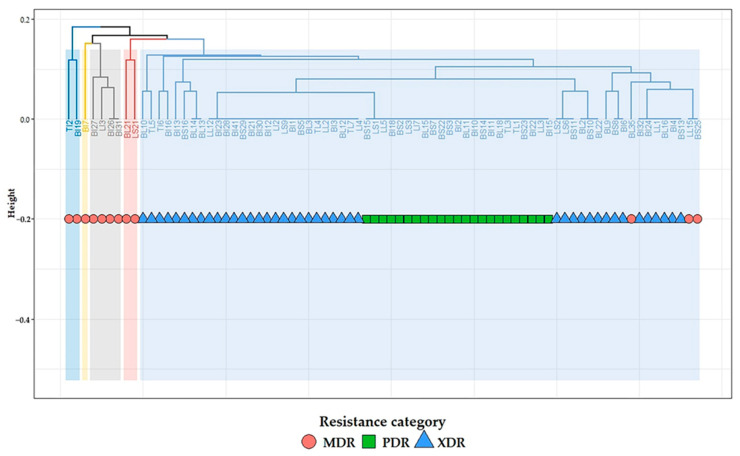
Hierarchical clustering dendrogram showing the relatedness of *C. perfringens* as determined by the antimicrobial resistance profiles. The code numbers refer to the isolates from broiler liver (BL), broiler spleen (BS), broiler intestine (BI), layer liver (LL), layer spleen (LS), layer intestine (LI), turkey intestine (TI), and turkey liver (TL) samples. MDR: multidrug-resistant; XDR: extensive drug-resistant; PDR: pan drug-resistant.

The clustering pattern of *C. perfringens* isolates is displayed in Figure 4. The examined *C. perfringens* isolates exhibited low diversity based on the antimicrobial resistance profiles. Among the 77 examined isolates, only 15 isolates belonged to various lineages. Two main clusters and five distinct subclusters were observed in our results, and close relatedness was determined between *C. perfringens* isolates from various sources (Figure 4).

### 2.3. Biofilm Formation by C. perfringens Isolates

**Figure 5:**
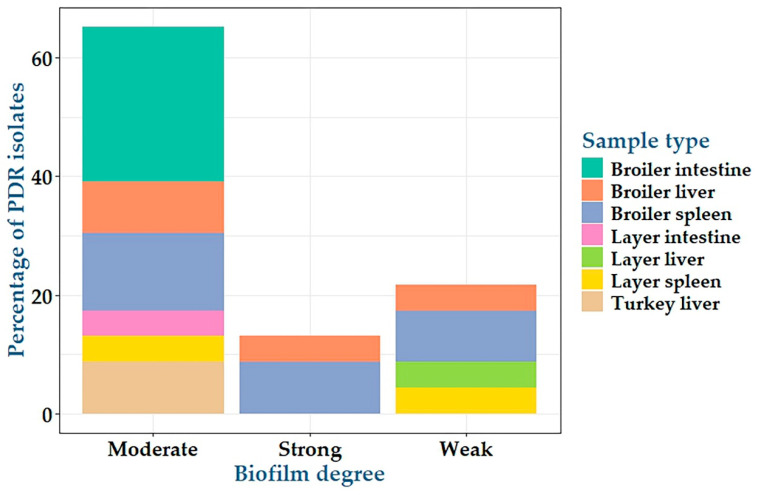
Differences in biofilm production abilities between pan drug-resistant *C. perfringens* isolates obtained from various sources.

Twenty-three PDR *C. perfringens* isolates were investigated for biofilm formation using the qualitative Congo red agar and the quantitative microtiter plate assays. The results showed that all tested *C. perfringens* isolates were biofilm producers; among them, 5, 15 and 3 isolates were weak, moderate, and strong biofilm producers with percentages of 21.7%, 65.2%, and 13.1%, respectively (Table 4, Figure 5, Supplementary Table S1 and Supplementary Figures S1 and S2). Among weak, moderate, and strong biofilm degrees, there were no statistically significant differences (*p* = 1, 0.166, and 1, respectively) between PDR *C. perfringens* isolates from various sources.

### 2.4. Antimicrobial Activity of CNCs/ZnO Bio-Nanocomposite Against PDR C. perfringens Isolates

**Figure 6:**
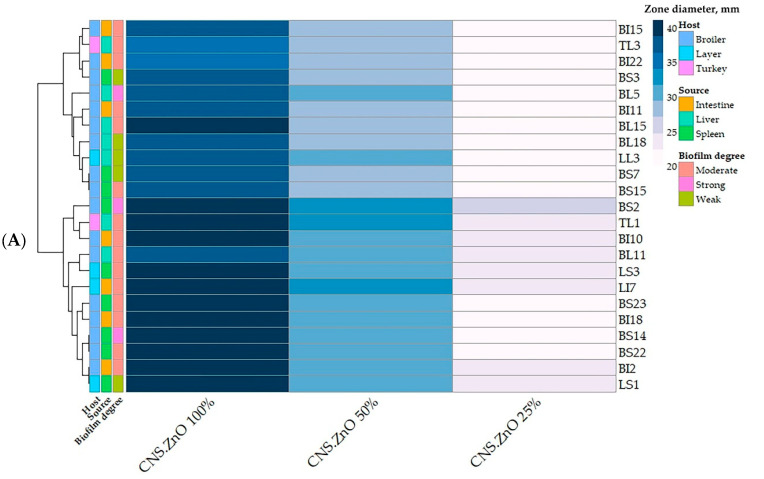

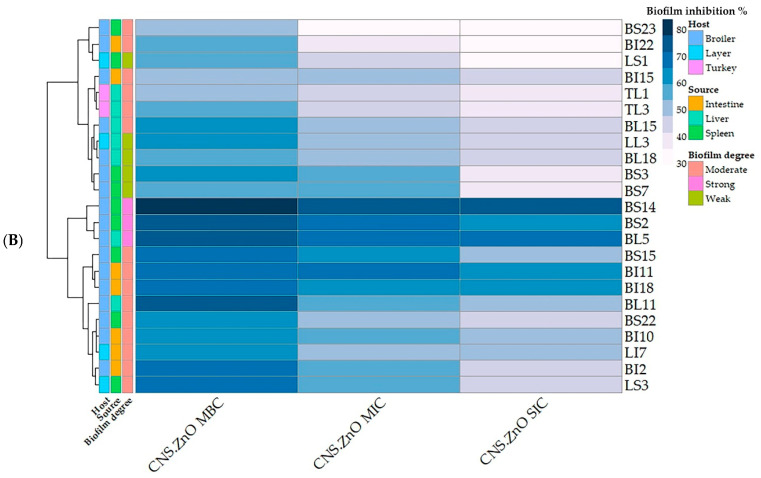
Heatmap showing the antimicrobial (**A**) and antibiofilm (**B**) activities of various concentrations of cellulose nanocrystal/ZnO (CNCs/ZnO) bio-nanocomposite against pan drug-resistant *C. perfringens* isolates. The scale on the right of the heatmap A refers to zone diameter in mm, and in heatmap B, it refers to biofilm inhibition%. The code numbers on the right of the heat map refer to the isolates from broiler liver (BL), broiler spleen (BS), broiler intestine (BI), layer liver (LL), layer spleen (LS), layer intestine (LI), and turkey liver (TL) samples. Different host and sample types are color-coded on the right of the heatmap.

The antimicrobial potentials of CNCs/ZnO bio-nanocomposite against PDR *C. perfringens* isolates were evaluated by determining the inhibition zones’ diameters and the minimum inhibitory concentration (MIC) values (Table 4 and Figure 6A). The results revealed that CNCs/ZnO bio-nanocomposite demonstrated marked inhibitory activity against *C. perfringens* with inhibition zones’ diameters of 36–40 mm at 100% concentration, 28–32 mm at 50% concentration and 20–25 mm at 25% concentration of the nanocomposite with an MIC value of 0.125 µg/mL (Supplementary Figure S3). Statistical analysis displayed significant differences in the antimicrobial activities among various concentrations of CNCs/ZnO bio-nanocomposite against 23 PDR *C. perfringens* isolates (*p* < 0.01). CNCs/ZnO bio-nanocomposite at 100% concentration showed the largest zone of inhibition in all tested isolates.

### 2.5. Antibiofilm Activity of CNCs/ZnO Bio-Nanocomposite Against PDR C. perfringens

Table 4 and Figure 6 display the antibiofilm activities of CNCs/ZnO bio-nanocomposite at their MBC, MIC, and SIC against 23 PDR *C. perfringens* isolates. The results showed that CNCs/ZnO exerted great activity against the pre-existent biofilms of PDR *C. perfringens* isolates in a dose-dependent manner ((MBIC50 up to 83.43 ± 1.98 for the CNS-ZnO MBC concentration (0.25 μg/mL)) (Supplementary Table S1 and Supplementary Figure S4). There were significant differences in the antibiofilm activities among MBC, MIC, and SIC of CNCs/ZnO bio-nanocomposite against the examined PDR *C. perfringens* isolates (*p* < 0.05) except isolates of code no. BI18 and BL5. CNCs/ZnO bio-nanocomposite at their MBC showed the highest biofilm inhibition% in 14 tested isolates. CNCs/ZnO bio-nanocomposite at their MIC and SIC showed the highest biofilm inhibition% in 7 tested isolates.

### 2.6. Expression Analysis of CNCs/ZnO Bio-Nanocomposite Against PDR C. perfringens Using RT-qPCR

**Figure 7:**
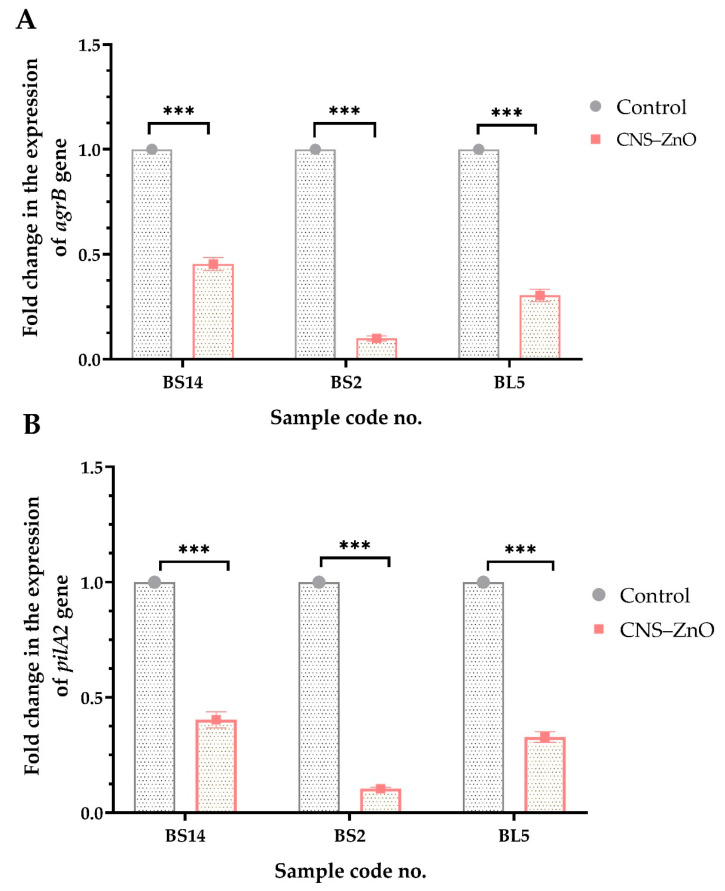
Fold changes in the expressions of *agrB* (**A**) and *pilA2* (**B**) genes among PDR *C. perfringens* isolates after treatment with cellulose nanocrystal/ZnO bio-nanocomposites (CNCs/ZnO). Values represent the fold changes in comparison with the transcription levels of the control untreated isolates, which were assigned a value of 1. Results were expressed as a means of three independent experiments ± standard error of the mean (SEM). *** *p* < 0.001. BS: broiler spleen, BL: broiler liver.

The expression of *agrB* quorum sensing gene and *pilA2* type IV pili biosynthesis gene responsible for biofilm formation were determined by the RT-qPCR technique pre- and post-treatment with the CNCs/ZnO nanocomposite. The results showed that the transcript levels of *agrB* and *pilA2* genes in CNCs/ZnO-treated biofilms decreased (0.099 ± 0.012–0.454 ± 0.031 and 0.104 ± 0.006–0.403 ± 0.035, respectively) when compared to the non-treated isolates. There were statistically significant differences in the transcriptional modulation of *agrB* and *pilA2* genes among CNCs/ZnO treated and control untreated *C. perfringens* isolates (*p* < 0.0001) (Figure 7).

## 3. Discussion

The poultry industry suffers significant financial losses due to NE, and resistant clostridial infections, particularly those caused by the PDR strains, could complicate the therapy [28]. Reducing the use of antibiotics in the animal industry is a significant worldwide concern because of their detrimental effects on public health [29]. This is the first report investigating the in vitro efficacy of the CNCs/ZnO bio-nanocomposite against PDR biofilm-producing *C. perfringens* accused of NE in Egypt.

Herein, the prevalence of *C. perfringens* was 44.4% in broilers, which matches with that reported in a previous study in Egypt (46%) [30] with the highest existence in the intestine. In comparison, Gomaa et al. [31] reported that the prevalence rate of *C. perfringens* in a chicken origin was 38.3% with a higher existence in the liver followed by muscle and intestine. The early colonization of *C. perfringens* in the digestive tract of poultry, even from hatchery, may be the cause of the high existence of the bacterium in chickens [32]. Also, the bacterium is a normal inhabitant of the chicken’s gut but may proliferate, causing a disease in certain conditions [33]. On the other hand, our result was lower than those reported by Ibrahim and co-authors [34], who isolated *C. perfringens* from the intestinal samples of diseased birds with a percentage of 80.9%. However, in a previous study [35], *C. perfringens* was identified in 38.37% of intestinal samples in six Egyptian Governorates. The low isolation rate of *C. perfringens* in intestinal samples may be attributed to the collection of samples from poultry farms exposed to antimicrobials for therapeutic purposes, which could destroy the gut microbial community in addition to geographical variations [35].

Globally, Zhang et al. [36] could isolate *C. perfringens* from fresh chicken meat in various outlets (13.6%) and poultry farms (23.1%) in China with lower percentages. Moreover, in Pakistan, Haider’s team [37] isolated *C. perfringens* from poultry farms with a prevalence rate of 25.37%. Similarly, Rana and coworkers [38] detected *C. perfringens* in chickens in Bangladesh with a percentage of 34.5%. In contrast, Yadav et al. [39] documented the overall prevalence rate of *C. perfringens* to be 66.8% and 25.6% among diseased birds with NE and healthy ones, respectively, in India.

In Egypt, NE in turkeys has become a significant economic issue since 2018, resulting in significant losses and raising serious concerns for breeders [40]. In the current study, the prevalence rate of *C. perfringens* was 58.3% in diseased turkeys. To date, few studies have investigated the prevalence of *C. perfringens* in turkeys. The occurrence of *C. perfringens* in this study nearly coincided with the findings of previously published documents [41] (55%) and [40] (41%). The limitations of this study should be acknowledged, including the relatively few numbers of available turkey farms in Egypt for sample collection, which constrained the sample size for this study and reduced the generalisability of the findings.

The variations in prevalence could be ascribed to various isolation techniques, sample selection (number and kind of samples from healthy and/or diseased birds), differences in geographical locations, management of chicken farms (presence or absence of NE predisposing conditions), and varying antibiotic dosing ranges.

Antimicrobials have been used in order to prevent NE and increase broiler production [42]. Antimicrobials are either used for therapeutic or preventative purposes in animal feed [43]. The use of antimicrobials in the poultry industry as growth promoters has contributed to antimicrobial resistance in bacteria [44]. However, this practice has since been banned in a number of countries [28]. The prolonged and extensive use of antimicrobials in poultry over the past few years has altered the bacterial environment, eliminated susceptible strains, and promoted the persistence and dominance of antimicrobial-resistant bacteria [11]. Since MDR strains have emerged as a result of antibiotic abuse, continuing to publish resistance rates is the first step in stopping the spread of antibiotic resistance [45]. In this study, we examined the susceptibilities of 77 *C. perfringens* isolates to 19 antimicrobials of 17 classes and offered greater insight into the concerning rise in PDR, XDR, and MDR profiles. Interestingly, 29.9%, 54.5%, and 15.6% of *C. perfringens* isolates were PDR, XDR, and MDR, respectively, with high MAR indices ranged from 0.84 to 1. A previous research [31] demonstrated the first record for the emergence of PDR *C. perfringens* isolate from a poultry source, while another study conducted on diseased and apparently healthy chickens [46] presented that eight isolates (26.7%) were XDR, one isolate (3.3%) was PDR, and twenty-one isolates (70%) were MDR. The increasing MAR indices suggest that the antimicrobials are being misused without restriction, either to treat clostridial infections in animals and poultry or as growth promoters to enhance growth performance. This promoted the development and dissemination of antibiotic resistance in the common intestinal flora, such as *C. perfringens* strains [47].

In addition to the ability of *C. perfringens* to produce toxins, it is able to form biofilms, which makes the bacteria resistant to disinfectants and antibiotics and contributes to its continuing existence in the environment [48,49]. Bacteria can adhere to substrates and encapsulate themselves in a matrix consisting of extracellular DNA (eDNA), proteins, and extracellular polymeric substances (EPS) when they form biofilms [15,16]. Their interactions with exopolysaccharides and nucleic acid constituents aid in the stability of the biofilm matrix, surface colonization, and preservation of the biofilm’s integrity and structure [18,50]. The biofilm layer gives the bacteria the ability to cause a variety of diseases. Thus, it is believed that biofilm-producing bacteria cause 65–80% of infections [51]. In addition to preserving a biofilm’s structural integrity, EPS acts as a barrier against antimicrobial substances, including antibiotics and bleaching chemicals [52].

In this study, all tested PDR *C. perfringens* isolates turned out to be biofilm producers, which matches a recently published work [53]. This indicates that the presence of slow-growing or persistent cells in biofilms [18] or increased expression of efflux pumps in biofilms [19,20] may be accused of antimicrobial resistance in biofilms.

In Egyptian poultry farms, antimicrobials are routinely administered from first day of life till slaughtering. This widespread and continuous use of antimicrobials creates a significant problem; each time an antimicrobial is used, the bacteria within the chickens’ systems have a higher chance of developing resistance. This resistance occurs because some bacteria possess genes that allow them to survive the antimicrobial effects, and these resistant bacteria then multiply, making future treatments less effective [54]. In this study, CNCs/ZnO nanocomposite showed strong inhibitory activity against *C. perfringens* isolates (inhibition zone diameters ranged from 36–41 mm for 100% concentration and MIC values ≤ 0.125 μg/mL. Moreover, CNCs/ZnO exerted great activity against the pre-existent biofilms of PDR *C. perfringens* isolates in a dose-dependent manner with the MBIC50 of up to 83.43 ± 1.98 for the CNCs/ZnO MBC concentration (0.25 μg/mL). Interestingly, there are no published issues regarding CNCs/ZnO nanoparticles’ antibacterial and antibiofilm properties against *C. perfringens* isolates to compare with the current findings. Significant antibacterial activities have been shown by the CNCs/ZnO nanocomposite against a variety of Gram-positive and Gram-negative bacteria, such as *Staphylococcus aureus*, *Salmonella*, and *Escherichia coli* [27]. The CNCs/ZnO nanocomposite can physically interact with bacterial cell walls, resulting in the destruction of its structure. This contact is facilitated by the nanocomposites’ high surface area and sharp edges, which rupture the membranes and allow cellular contents to leak out [27,55]. Another important mechanism of action of the CNC/ZnO nanocomposite is oxidative stress [55,56]. When ZnO nanoparticles are exposed to light, they produce reactive oxygen species (ROS), which can harm the lipids, proteins, and DNA of bacterial cells. Moreover, the CNC/ZnO nanocomposite can block important bacterial enzymes that are essential for bacterial survival and replication, including dihydrofolate reductase (DHFR) and dihydropteroate synthase (DHPS). This enzymatic inhibition further contributes to the antibacterial properties of the nanocomposites [56,57].

CNC/ZnO bio-nanocomposite has demonstrated antibiofilm activities by interfering with the extracellular polymeric materials that hold biofilms together or penetrating the biofilms and exhibiting antibacterial properties within the biofilm matrix; thus, the bio-nanocomposite can decrease the viability of bacteria entrenched in biofilms [27,58].

In the present study, the expression of *agrB* quorum sensing and *pilA2* type IV pili biosynthesis gene responsible for biofilm formation were inspected by the RT-qPCR technique before and after treatment with the CNCs/ZnO. The relative expression of *agrB* and *pilA2* genes in CNCs/ZnO-treated biofilms was significantly reduced than the non-treated ones. However, similar data were not available for comparison with other authors about the antibiofilm activity of CNCs/ZNO against PDR *C. perfringens* bacterial pathogens.

## 4. Materials and Methods

### 4.1. Samples and Ethical Approval

Poultry samples, including intestines, livers, and spleens, were collected from 172 birds (three samples from each bird) comprising chickens (broilers (n = 124) and layers (n = 36)) and turkeys (n = 12) from various poultry farms in Egypt during the period between November 2022 and June 2023. The birds were clinically examined for any observable clinical signs and gross lesions related to NE. They mostly suffered from diarrhea, low growth rate, and mortality. The samples were transported immediately in an ice box to the bacteriology laboratory for further analysis. The animal study was approved by the Institutional Animal Care and Use Committee, Faculty of Veterinary Medicine, Zagazig University (Approval No ZU-IACUC/2/F/149/2024).

### 4.2. Bacteriological Analysis and Molecular Identification

*Clostridium perfringens* were isolated under anaerobic conditions in accordance with the previously defined technique [59]. The samples were enriched for 24 h at 37 °C in cooked meat media (Oxoid, Cambridge, UK). A loopful of enrichment broth culture was plated onto Perfringens Agar Base (Oxoid, Cambridge, UK) provided with TSC supplement (D-cycloserine 200 mg/vial) and egg yolk emulsion then incubated at 37 °C for 24 h. Colonies of presumed *C. perfringens* isolates were verified by target hemolysis on blood agar, lecithinase activity, motility test, and skim milk coagulation (stormy fermentation) [60]. For molecular confirmation of bacterial isolates, the bacterial DNAs were extracted using a QIAamp DNA Mini kit (Qiagen GmbH, Hilden, Germany) following the manufacturer’s instructions. The *16S rRNA* gene of *C. perfringens* was amplified by conventional PCR using the oligonucleotide primer pair listed in Supplementary Table S2 [61].

### 4.3. Antimicrobial Susceptibility Testing

Antimicrobial susceptibility testing of *C. perfringens* isolates against 19 antimicrobial discs (Oxoid, Cambridge, UK) within 17 different categories was carried out adopting the standard disc diffusion protocol following the Clinical and Laboratory Standards Institute guidelines [62]. The antimicrobials included penicillin (penicillin G (P; 1 unit)), penicillin combinations (amoxycillin-clavulanic acid (AMC; 20/10 µg)), cephalosporines (cefoxitin (FOX; 30 µg)), carbapenems (imipenem (IPM; 10 µg)), aminoglycosides (gentamicin (CN; 10 µg)), macrolides (erythromycin (E; 15 µg)), quinolones (nalidixic acid (NA; 30 µg)), fluoroquinolones (enrofloxacin (ENR; 5 µg)), sulfonamides (sulfamethoxazole-trimethoprim (SXT; 23.75/1.25 μg)), amphenicols (chloramphenicol (C; 30 µg)), polypeptides (bacitracin (B; 10 µg)), oxazolidinones (linezolid (LNZ; 30 µg)), lincosamides (clindamycin (DA; 2 µg) and lincomycin (L2; 15 μg)), tetracyclines (tetracycline (TE; 30 µg)), glycopeptides (vancomycin (VA; 30 µg) and teicoplanin (Tec; 30 µg)), nitroimidazole (metronidazole (MET; 5 µg)), and rifamycin (rifampin (RA; 5 µg)). Based on previous reports [31,63] for the majority of antimicrobials and the British Society for Antimicrobial Chemotherapy (BSAC) for penicillin G, imipenem, clindamycin, and metronidazole [64], the interpretive criteria of antimicrobial resistance were established. The multiple antimicrobial resistance (MAR) indices were determined as reported elsewhere [65]. The following drug resistance categories were identified as previously reported [66]: MDR; resistance to three or more classes of antimicrobial agents, XDR; resistance to all classes of antimicrobial agents except two or fewer, and PDR; resistance to all antimicrobial agents. A *C. perfringens* ATCC 13124 strain was used as quality control.

### 4.4. Biofilm Growth and Quantification

#### 4.4.1. Qualitative Congo Red Agar Method

*Clostridium perfringens* isolates were incubated on brain heart infusion agar (BHI; Oxoid, UK) supplemented with 5% (*w*/*v*) sucrose and 0.08% (*w*/*v*) Congo red dye (Oxoid, Hampshire, UK) for 24 to 48 h at 37 °C. Black colonies with dry crystalline phenotype indicated biofilm formation, while weak or non-biofilm producing isolates formed pink-coloured colonies [67].

#### 4.4.2. Quantitative Microtiter Plate Assay

Biofilm production by *C. perfringens* isolates was detected using sterile 96-well flat-bottomed polystyrene microtiter plates (Techno Plastic Products, Trasadingen, Switzerland). In brief, 200 μL of 10^6^ CFU/mL bacterial suspension was inoculated in each well and then incubated for 24 h at 37 °C. Negative control wells containing 200 µL of uninoculated Tryptic Soy Broth (TSB; Oxoid, Cambridge, UK) were included. The wells were gently washed using 200 μL of phosphate-buffered saline (PBS) three times, then dried with their sides facing up for 15 min [68]. To stain the biofilm mass, 50 μL of 0.1% (*w*/*v*) crystal violet (Oxoid, UK) was added for 30 min; the wells were washed three times with 200 μL of PBS and then air dried upside-down. To solubilize the stain, the wells were finally dissolved in 200 μL of ethanol/acetone solution (80:20, *v*/*v*). The optical density (OD) of biofilm mass was measured at 570 nm using a microplate reader (Stat Fax 2100, Awareness Technology, Palm City, FL, USA). The OD cut-off (ODc) was defined as three standard deviations over the mean OD of the negative control. The isolates were categorized into four groups based on their adhesion capabilities: non-biofilm producers (OD ≤ ODc), weak biofilm producers (ODc < OD ≤ 2xODc), moderate biofilm producers (2ODc < OD ≤ 4xODc), and strong biofilm producers (4xODc < OD) [69,70].

### 4.5. Synthesis of CNCs/ZnO Bio-Nanocomposite

The CNCs/ZnO bio-nanocomposite was synthesized following Dawwam et al. [27] at the Institute of Nanoscience and Nanotechnology, Kafrelsheikh University, Kafr el-Sheikh, Egypt. Briefly, 3.5 g zinc acetate was added to 1% cellulose nanocrystal (CNC) suspension, extracted from Palm sheath fibers. The mixture was stirred (30 min), sonicated (2 h, 80 °C or 35 °C), dialyzed (conductivity ≈ 4 μS·cm⁻^1^), and dried (60 °C, 6 h). Characterization of the nanocomposite was applied, as illustrated in the previously published work [27].

### 4.6. Antimicrobial Activities of CNCs/ZnO Against PDR C. perfringens

For the agar well diffusion technique, colonies from an overnight culture of *C. perfringens* were standardized to 1 × 10^8^ CFU. Each isolate was swabbed twice on a sterile Muller-Hinton agar plate. By using a sterile 1 mL pipette tip, multiple wells were created (approximately 6 mm in diameter), then 100 µL of CNCs/ZnO bio-nanocomposite was inoculated into the wells at different concentrations (10, 50, and 100%). Sterile distilled water was used as a negative control, while the imipenem antibiotic was a positive control. The plates were incubated anaerobically at 37 °C for 24 h, and then zones of growth inhibition were measured to the nearest millimeter. The isolates with inhibition zones diameter ≥ 8 mm were considered susceptible [71].

The minimum inhibitory concentration (MIC) and minimum bactericidal concentration (MBC) of CNCs/ZnO bio-nanocomposite were then determined using the broth microdilution method [72]. Briefly, two-fold serial dilutions of CNCs/ZnO were performed from the stock solution (512 μg/mL), then 100 μL of each dilution was dispended in 96-well culture plates. Thereafter, 100 μL of bacterial suspension of approximately 5 × 10^5^ CFU/mL was added to each well and incubated under anaerobic conditions at 37 °C for 24 h. Ten µL of resazurin dye (stock preparation; 337.5 mg of resazurin powder in 50 mL sterile distilled water) was added to each well; then incubation was done at 37 °C for 2–4 h. The color change is based on bacterial metabolic activity; wells with no bacterial growth remain blue, while wells with growth turn pink [73]. The lowest concentration of CNCs/ZnO exhibiting no growth was considered as the MIC, whereas that which kills 99.9% of bacteria was considered the MBC.

### 4.7. Antibiofilm Activities of CNCs/ZnO Bio-Nanocomposite Against Biofilm Producing C. perfringens

This assay investigated the ability of CNCs/ZnO bio-nanocomposite to disperse biofilm formation by *C. perfringens*. Briefly, 100 µL of the antibacterial agent at different concentrations (512–0.25 µg/mL) was added to selected wells of 96-well flat-bottom polystyrene microtiter plate seeded with 100 µL of the *C. perfringens* suspension (10^8^ cells/mL) and incubated for 24 h at 37 °C to allow biofilm formation. The contents of the wells were aspirated and washed three times with sterile PBS. The extent of biofilm formation was assessed using the crystal violet staining assay as described previously [65,66]. The negative controls (CNCs/ZnO free wells) and the positive controls (*C. perfringens* biofilm producers) were included. Experiments were carried out in triplicate [74]. The inhibition percentage of biofilm was calculated by the formula: Percentage of biofilm inhibition = (Control OD_570_ nm − Test OD_570_ nm)/Control OD_570_ nm) × 100 [75,76]. Further, the minimal biofilm inhibitory concentration (MBIC) was defined as the minimal antimicrobial concentration showing no color development.

### 4.8. Gene Expression of Biofilm Biosynthesis Genes by Real-Time Quantitative PCR (RT-qPCR)

Pan drug-resistant strong biofilm producers *C. perfringens* isolates were separately exposed to the subinhibitory concentration (SIC) of the CNCs/ZnO bio-nanocomposite and then incubated at 37 °C for 24 h. Non-treated *C. perfringens* biofilms were considered controls. Biofilms were harvested and then gently washed with PBS to remove the planktonic cells. Total RNAs were extracted from biofilms of both treated and untreated *C. perfringens* isolates using a QIAampRN easy Mini kit (Qiagen, Hilden, Germany) following the manufacturer’s recommendations. Relative expressions of the biofilm biosynthesis gene (*pilA2*) and its regulator (*agrB*) were investigated by one-step RT-qPCR using the QuantiTect SYBR Green RT-PCR kit (Qiagen, Germany) in the MX3005p real-time PCR thermal cycler (Stratagene, Lajolla, CA, USA) according to the manufacturer’s guidelines using oligonucleotide primers listed in Supplementary Table S2) [77,78]. The *16S rRNA* housekeeping gene was used for normalization [79]. Amplification curves and threshold cycle (c_t_) values of tested isolates were determined to be compared with the control positive according to the “ΔΔC_t_” method stated previously [80] using the following ratio: (2 − ΔΔC_t_).

### 4.9. Statistical Analysis

The data were analyzed using SPSS version 26 (IBM Corp, Armonk, NY, USA). The Chi-square test was used to study the variations in the prevalence of *C. perfringens* from different origins and to assess the differences in the antimicrobial resistance patterns of the recovered isolates from various sources. Additionally, one-way ANOVA and Tukey’s post hoc test were used to evaluate the antibiofilm and the antibacterial efficacy of CNCs/ZnO bio-nanocomposite at various concentrations against PDR *C. perfringens* isolates. All experimental procedures were done in triplicate, and the results were expressed as mean ± standard error of the mean (SEM). The *p*-values were considered statistically significant if they were less than 0.05. All graphs were generated by R-software version 4.4.3 [81] (https://www.r-project.org/) using ggplot [82], pheatmap [83], and factoextra [84] packages. Moreover, we used an independent samples *t*-test to test CNCs/ZnO effects on *C. perfringens* biofilm genes’ expression. All tests were done using SPSS Inc. version 26 (IBM Corp., Armonk, NY, USA). The *p*-values of less than 0.05 were considered statistically significant. Figures were generated by GraphPad Prism version 8 (San Diego, CA, USA).

## 5. Conclusions

To the best of our knowledge, this is the first report demonstrating the antimicrobial and antibiofilm activities of CNCs/ZnO nanocomposite against PDR *C. perfringens* isolates recovered from chickens and turkeys and their pre-existent biofilms, providing a distinct benefit over the conventional antimicrobial agents.

## Figures and Tables

**Table 1:** Prevalence of *C. perfringens* in poultry samples.

Source (No.)	Prevalence of *C. perfringens* No. (%) ^a^	*p*-Value ^b^
Broiler chicken (124)	55 (44.35)	
Liver	15 (12.1)	0.122
Spleen	15 (12.1)	
Intestine	25 (20.16)	
Layer (36)	15 (41.67)	
Liver	5 (13.89)	1
Spleen	6 (16.67)	
Intestine	4 (11.11)	
Turkey (12)	7 (58.33)	
Liver	5 (41.67)	0.046
Spleen	0 (0.0)	
Intestine	2 (16.67)	
*p*-value ^c^	-	0.608
Total (172)	77 (44.77)	-

^a^ The isolation rate was calculated concerning the total number of the examined samples from each source. ^b^ *p*-value among liver, spleen, and intestine isolates from each host. ^c^ *p*-value among total broiler, layer, and turkey isolates.

**Table 2:** Antibiogram of *C. perfringens* isolates recovered from poultry samples.

Antimicrobial Class	AMA	No. of Resistant Isolates (%)	MAR Index	*p*-Value
Penicillin	Penicillin G benzylpenicillin	74 (96.1)	0.051	<0.0001 ***
Penicillin combinations	Amoxycillin-clavulanic acid	76 (98.7)	0.052	<0.0001 ***
Cephalosporins	Cefoxitin	71 (92.2)	0.049	<0.0001 ***
Carbapenems	Imipenem	39 (50.65)	0.026	0.374
Aminoglycosides	Gentamicin	75 (97.1)	0.051	<0.0001 ***
Macrolides	Erythromycin	77 (100)	0.053	NA
Quinolones	Nalidixic acid	76 (98.7)	0.052	<0.0001 ***
Fluoroquinolone	Enrofloxacin	76 (98.7)	0.052	<0.0001 ***
Sulfonamides	Sulfamethoxazole-trimethoprim	58 (75.32)	0.040	<0.0001 ***
Amphenicols	Chloramphenicol	64 (83.12)	0.044	<0.0001 ***
Polypeptides	Bacitracin	77 (100)	0.053	NA
Oxazolidones	Linezolid	69 (89.61)	0.047	<0.0001 ***
Lincosamides	Clindamycin	77 (100)	0.053	NA
	Lincomycin	77 (100)	0.053	NA
Tetracyclines	Tetracycline	77 (100)	0.053	NA
Glycopeptides	Vancomycin	77 (100)	0.053	NA
	Teicoplanin	77 (100)	0.053	NA
Nitroimidazole	Metronidazole	74 (96.1)	0.051	<0.0001 ***
Antimycobacterials	Rifampin	77 (100)	0.053	NA

AMA: antimicrobial agent; MAR: multiple antimicrobial resistance; NA: non-applicable; *** *p* < 0.001.

**Table 3:** Frequency of resistance to various antimicrobial agents and classes in *C. perfringens* isolates belonging to various sources.

MAR Index	Resistance to AMA (n = 19)	Resistance to AMC (n = 17)	No. of Resistant Isolates (%)			*p*-Value	Total No. of Isolates (%) (n = 77)	Resistance Category
			Broiler (n = 55)	Layer (n = 15)	Turkey (n = 7)			
0.79	15	13	2 (3.63)	1 (6.67)	0	1	4 (5.19)	MDR
0.84	16	14	6 (10.91)	2 (13.33)	1 (14.29)	1	8 (10.39)	
0.89	17	15	10 (18.18)	3 (20)	1 (14.29)	1	14 (18.18)	XDR
0.95	18	16	20 (36.36)	5 (33.33)	3 (42.86)	1	28 (36.36)	
1	19	17	17 (30.91)	4 (26.67)	2 (28.57)	1	23 (29.87)	PDR

MAR: multiple antimicrobial resistance; AMA: antimicrobial agent; AMC: antimicrobial class; MDR: multidrug-resistant; XDR: extensive drug-resistant; PDR: pan drug-resistant.

**Table 4:** Biofilm production, antimicrobial and antibiofilm activities of cellulose nanocrystal/ZnO bio-nanocomposite against pan drug-resistant *C. perfringens* isolates.

Code No.	PDR Isolate No.	Source	OD570	Biofilm Degree	CNCs/ZnO Activity (Zone Diameter, mm)			*p*-Value	Biofilm Inhibition % at			*p*-Value
					100%	50%	25%		MBC	MIC	SIC	
LL3	1	Layer liver	0.352	Weak	38 ± 1.15 ^a^	30 ± 0.58 ^b^	21 ± 0.58 ^c^	<0.0001 ***	59.37 ± 1.95 ^a^	47.51 ± 1.45 ^b^	41.98 ± 1.14 ^b^	0.001 **
BI15	2	Broiler intestine	0.542	Moderate	37 ± 1.73 ^a^	28 ± 1.15 ^b^	20 ± 1.15 ^c^	<0.0001 ***	52.95 ± 1.7 ^a^	48.70 ± 2.14 ^a b^	42.98 ± 1.72 ^b^	0.025 *
BI22	3	Broiler intestine	0.449	Moderate	36 ± 2.31 ^a^	28 ± 0.58 ^b^	21 ± 1.15 ^c^	0.001 **	57.68 ± 1.55 ^a^	36.08 ± 0.62 ^b^	34.07 ± 1.2 ^b^	<0.0001 ***
BS23	4	Broiler spleen	0.422	Moderate	40 ± 1.73 ^a^	31 ± 1.15 ^b^	22 ± 0.58 ^c^	<0.0001 ***	53.08 ± 1.78 ^a^	30.09 ± 1.15 ^b^	29.14 ± 0.66 ^b^	<0.0001 ***
TL1	5	Turkey liver	0.416	Moderate	41 ± 0.58 ^a^	32 ± 1.75 ^b^	23 ± 1.15 ^c^	<0.0001 ***	52.40 ± 1.39 ^a^	44.71 ± 2.14 ^b^	39.90 ± 1.1 ^b^	0.004 **
TL3	6	Turkey liver	0.410	Moderate	36 ± 0.58 ^a^	28 ± 0.58 ^b^	20 ± 0.33 ^c^	<0.0001 ***	54.39 ± 1.38 ^a^	46.34 ± 0.77 ^b^	38.29 ± 1.32 ^c^	<0.0001 ***
BL18	7	Broiler liver	0.330	Weak	37 ± 1.15 ^a^	29 ± 0.58 ^b^	21 ± 0.58 ^c^	<0.0001 ***	57.57 ± 1.48 ^a^	47.57 ± 1.48 ^b^	42.42 ± 1.4 ^b^	0.001 **
BI11	8	Broiler intestine	0.706	Moderate	38 ± 1.73 ^a^	29 ± 1.15 ^b^	20 ± 1.15 ^c^	<0.0001 ***	70.11 ± 1.15 ^a^	66.43 ± 0.83 ^a^	60.19 ± 1.73 ^b^	0.005 **
BS14	9	Broiler spleen	0.851	Strong	39 ± 0.58 ^a^	30 ± 1.15 ^b^	22 ± 1.15 ^c^	<0.0001 ***	83.43 ± 1.98 ^a^	76.96 ± 1.71 ^a b^	74.38 ± 1.73 ^b^	0.03 *
BI10	10	Broiler intestine	0.608	Moderate	41 ± 0.58 ^a^	31 ± 1.73 ^b^	24 ± 0.58 ^c^	<0.0001 ***	62.66 ± 1.15 ^a^	53.94 ± 1.73 ^b^	47.36 ± 1.15 ^c^	0.001
BL11	11	Broiler liver	0.551	Moderate	38 ± 1.73 ^a^	30 ± 1.15 ^b^	24 ± 1.15 ^b^	0.002 **	76.04 ± 0.6 ^a^	55.17 ± 0.68 ^b^	50.63 ± 1.37 ^c^	<0.0001 ***
BI2	12	Broiler intestine	0.401	Moderate	39 ± 1.15 ^a^	30 ± 1 ^b^	23 ± 1.73 ^c^	<0.0001 ***	66.58 ± 1.15 ^a^	55.11 ± 1.22 ^b^	44.38 ± 1.73 ^c^	<0.0001 ***
BS3	13	Broiler spleen	0.321	Weak	37 ± 1.73 ^a^	28 ± 0.58 ^b^	21 ± 1.15 ^c^	<0.0001 ***	60.74 ± 1.58 ^a^	55.76 ± 1.59 ^a^	38.94 ± 1.15 ^b^	<0.0001 ***
BS22	14	Broiler spleen	0.429	Moderate	39 ± 1.73 ^a^	30 ± 1.15 ^b^	22 ± 1.15 ^c^	<0.0001 ***	63.63 ± 1.73 ^a^	51.04 ± 0.6 ^b^	46.38 ± 1.73 ^b^	<0.0001 ***
BS7	15	Broiler spleen	0.290	Weak	38 ± 1.15 ^a^	29 ± 0.58 ^b^	21 ± 1.73 ^b^	<0.0001 ***	55.17 ± 1.15 ^a^	53.79 ± 1.73 ^a^	39.65 ± 0.95 ^b^	<0.0001 ***
BL15	16	Broiler liver	0.473	Moderate	39 ± 0.58 ^a^	29 ± 0.58 ^b^	20 ± 0.58 ^c^	<0.0001 ***	61.94 ± 1.12 ^a^	51.37 ± 0.79 ^b^	42.91 ± 1.15 ^c^	<0.0001 ***
LI7	17	Layer intestine	0.447	Moderate	39 ± 0.58 ^a^	32 ± 1.73 ^b^	23 ± 1.15 ^c^	<0.0001 ***	65.10 ± 1.21 ^a^	53.02 ± 1.73 ^b^	47.87 ± 1.66 ^b^	0.001 **
LS3	18	Layer spleen	0.655	Moderate	39 ± 0.58 ^a^	31 ± 0.15 ^b^	24 ± 0.58 ^c^	<0.0001 ***	69.46 ± 1.42 ^a^	57.25 ± 1.3 ^b^	46.10 ± 0.64 ^c^	<0.0001 ***
BS2	19	Broiler spleen	0.739	Strong	40 ± 1.15 ^a^	32 ± 1.15 ^b^	25 ± 1.15 ^c^	<0.0001 ***	75.64 ± 1.73 ^a^	68.74 ± 1.58 ^a b^	63.05 ± 1.73 ^b^	0.005 **
BI18	20	Broiler intestine	0.528	Moderate	40 ± 0.58 ^a^	30 ± 1.15 ^b^	22 ± 1.15 ^c^	<0.0001 ***	66.85 ± 1.12	62.87 ± 1.66	60.60 ± 1.5	0.086
BL5	21	Broiler liver	0.780	Strong	38 ± 1.15 ^a^	31 ± 0.58 ^b^	20 ± 0.58 ^c^	<0.0001 ***	73.07 ± 1.73	70.76 ± 1.59	67.94 ± 1.7	0.176
LS1	22	Layer spleen	0.280	Weak	39 ± 0.58 ^a^	30 ± 1.73 ^b^	23 ± 1.15 ^c^	<0.0001 ***	53.57 ± 2.06 ^a^	42.85 ± 1.65 ^b^	33.57 ± 1.48 ^c^	0.001 **
BS15	23	Broiler spleen	0.403	Moderate	38 ± 1.15 ^a^	29 ± 1.58 ^b^	21 ± 0.58 ^c^	<0.0001 ***	66.74 ± 1.58 ^a^	64.01 ± 1.15 ^a^	52.85 ± 1.12 ^b^	<0.0001 ***

PDR: pan drug-resistant; OD: optical density; CNCs/ZnO: cellulose nanocrystal/ZnO bio-nanocomposites. Results are expressed as means of triplicate experiment ± standard error of the mean (SEM). ^a–c^ Mean values with different superscript letters within the same row represent statistical significance (*p* > 0.05). * *p* < 0.05, ** *p* < 0.01, and *** *p* < 0.001.

## Data Availability

The data presented in this study are available upon request from the corresponding author.

## Supplementary Materials

The following supporting information can be downloaded at: https://www.mdpi.com/article/10.3390/antibiotics14060575/s1, Supplementary Figure S1: PDR *C. perfringens* isolates on Congo red agar media showing strong (A), Moderate (B) and weak (C) biofilm formation. Supplementary Figure S2: Biofilm formation by *C. perfringens* isolates using the crystal violet microtiter plate biofilm assay. A: An inoculated plate by 23 PDR *C. perfringens* isolates. B: Stained biofilms of PDR *C. perfringens* isolates by 0.1% crystal violet. C: *C. perfringens* biofilms after washing the stain using phosphate buffer saline. Supplementary Figure S3: Antimicrobial activity of CNCs/ZNO against PDR *C. perfringens* isolates. A: Agar well diffusion of CNCs/ZnO against a *C. perfringens* isolate. B: Broth microdilution method for determining the minimum inhibitory concentrations of CNCs/ZnO against *C. perfringens* isolates using Resazurin dye. Supplementary Figure S4: Antibiofilm activity of CNCs/ZnO bio-nanocomposite against 23 PDR *C. perfringens* isolates. A: An inoculated plate by 23 PDR *C. perfringens* isolates were then exposed to the nanocomposite. B: Stained biofilms of PDR *C. perfringens* isolates by 0.1% crystal violet. C: *C. perfringens* biofilms after washing the stain using phosphate buffer saline. Supplementary Table S1: Biofilm production by PDR *C. perfringens* isolates and the antibiofilm activities of CNCs/ZnO bio-nanocomposites using the microtiter plate method. Supplementary Table S2: Oligonucleotide primers used in the study.

## References

[1] Mottet A., Tempio G. (2017). Global poultry production: Current state and future outlook and challenges. World’s Poult. Sci. J..

[2] Timbermont L., Haesebrouck F., Ducatelle R., Van Immerseel F. (2011). Necrotic enteritis in broilers: An updated review on the pathogenesis. Avian Pathol..

[3] Cooper K.K., Songer J.G., Uzal F.A. (2013). Diagnosing clostridial enteric disease in poultry. J. Vet. Diagn. Investig..

[4] Fathima S., Hakeem W.G.A., Shanmugasundaram R., Selvaraj R.K. (2022). Necrotic enteritis in broiler chickens: A review on the pathogen, pathogenesis, and prevention. Microorganisms.

[5] Gharib-Naseri K., Kheravii S., Keerqin C., Morgan N., Swick R., Choct M., Wu S.-B. (2019). Two different *Clostridium perfringens* strains produce different levels of necrotic enteritis in broiler chickens. Poult. Sci..

[6] Rood J.I., Adams V., Lacey J., Lyras D., McClane B.A., Melville S.B., Moore R.J., Popoff M.R., Sarker M.R., Songer J.G. (2018). Expansion of the *Clostridium perfringens* toxin-based typing scheme. Anaerobe.

[7] Sattar M.M.K., Anjum A.A., Chang Y.F., Yaqub T., Aslam A., Ali T. (2023). Molecular characterization and toxins optimization of indigenous *Clostridium perfringens* toxinotype B isolated from lamb dysentery clinical cases. Kafkas Univ. Vet. Fak. Derg..

[8] de Souza M., Baptista A.A.S., Menck-Costa M.F., Justino L., da Glória E.M., Shimizu G.D., Ferraz C.R., Verri W.A., Van Immerseel F., Bracarense A. (2024). Modulation of broiler intestinal changes induced by *Clostridium perfringens* and deoxynivalenol through probiotic, paraprobiotic, and postbiotic supplementation. Toxins.

[9] Aqeel M., Mirani A.H., Khoso P.A., Sahito J.K., Bhutto A.L., Leghari R.A., Rahimoon M.M., Ali K., Ali N. (2024). A review on the study of immunomodulators and herbal remedies: A natural approach to treating necrotic enteritis. Pure Appl. Biol. (PAB).

[10] El-Aziz N.K.A., Tartor Y.H., Gharieb R.M.A., Erfan A.M., Khalifa E., Said M.A., Ammar A.M., Samir M. (2021). Extensive drug-resistant *Salmonella enterica* Isolated from poultry and humans: Prevalence and molecular determinants behind the co-resistance to ciprofloxacin and tigecycline. Front. Microbiol..

[11] Góchez D., Raicek M., Pinto Ferreira J., Jeannin M., Moulin G., Erlacher-Vindel E. (2019). OIE annual report on antimicrobial agents intended for use in animals: Methods used. Front. Vet. Sci..

[12] Ray S., Löffler S., Richter-Dahlfors A. (2024). High-resolution large-area image analysis deciphers the distribution of *Salmonella* cells and ECM components in biofilms formed on charged PEDOT: PSS surfaces. Adv. Sci..

[13] Pantaléon V., Bouttier S., Soavelomandroso A.P., Janoir C., Candela T. (2014). Biofilms of *Clostridium* species. Anaerobe.

[14] Kelly S.M., Lanigan N., O’Neill I.J., Bottacini F., Lugli G.A., Viappiani A., Turroni F., Ventura M., van Sinderen D. (2020). *Bifidobacterial* biofilm formation is a multifactorial adaptive phenomenon in response to bile exposure. Sci. Rep..

[15] Yu S., Su T., Wu H., Liu S., Wang D., Zhao T., Jin Z., Du W., Zhu M.-J., Chua S.L. (2015). PslG, a self-produced glycosyl hydrolase, triggers biofilm disassembly by disrupting exopolysaccharide matrix. Cell Res..

[16] Czuban M., Srinivasan S., Yee N.A., Agustin E., Koliszak A., Miller E., Khan I., Quinones I., Noory H., Motola C. (2018). Bio-orthogonal chemistry and reloadable biomaterial enable local activation of antibiotic prodrugs and enhance treatments against *Staphylococcus aureus* infections. ACS Cent. Sci..

[17] De Oliveira A., Cataneli Pereira V., Pinheiro L., Moraes Riboli D.F., Benini Martins K., Ribeiro de Souza da Cunha M.d.L. (2016). Antimicrobial resistance profile of planktonic and biofilm cells of *Staphylococcus aureus* and coagulase-negative staphylococci. Int. J. Mol. Sci..

[18] Li P., Yin R., Cheng J., Lin J. (2023). Bacterial biofilm formation on biomaterials and approaches to its treatment and prevention. Int. J. Mol. Sci..

[19] Seukep A.J., Mbuntcha H.G., Kuete V., Chu Y., Fan E., Guo M.-Q. (2022). What Approaches to thwart bacterial efflux pumps-mediated resistance?. Antibiotics.

[20] Gaurav A., Bakht P., Saini M., Pandey S., Pathania R. (2023). Role of bacterial efflux pumps in antibiotic resistance, virulence, and strategies to discover novel efflux pump inhibitors. Microbiology.

[21] Dovnar R., Smotryn S., Anufrik S., Anuchin S., Dovnar I., Iaskevich N. (2022). Copper and selenium nanoparticles as a new tool against antibiotic-resistant pathogenic microorganisms. Surg. East. Eur..

[22] Hashem A.H., Al-Askar A.A., Haponiuk J., Abd-Elsalam K.A., Hasanin M.S. (2023). Biosynthesis, characterization, and antifungal activity of novel trimetallic copper oxide–selenium–zinc oxide nanoparticles against some *mucorales* fungi. Microorganisms.

[23] Raja F.N., Worthington T., Martin R.A. (2023). The antimicrobial efficacy of copper, cobalt, zinc and silver nanoparticles: Alone and in combination. Biomed. Mater..

[24] Li H., Yang Z., Khan S.A., Walsh L.J., Seneviratne C.J., Ziora Z.M. (2024). Characteristics of metallic nanoparticles (especially silver nanoparticles) as anti-biofilm agents. Antibiotics.

[25] Terea H., Rebiai A., Selloum D., Tedjani M.L. (2024). Cellulose/ZnO nanoparticles (CNC/ZnO NPs): Synthesis, characterization, and evaluation of their antibacterial and antifungal activities. Cellulose.

[26] Kim I., Viswanathan K., Kasi G., Thanakkasaranee S., Sadeghi K., Seo J. (2022). ZnO nanostructures in active antibacterial food packaging: Preparation methods, antimicrobial mechanisms, safety issues, future prospects, and challenges. Food Rev. Int..

[27] Dawwam G.E., Al-Shemy M.T., El-Demerdash A.S. (2022). Green synthesis of cellulose nanocrystal/ZnO bio-nanocomposites exerting antibacterial activity and downregulating virulence toxigenic genes of food-poisoning bacteria. Sci. Rep..

[28] García-Vela S., Martínez-Sancho A., Said L.B., Torres C., Fliss I. (2023). Pathogenicity and antibiotic resistance diversity in *Clostridium perfringens* isolates from poultry affected by necrotic enteritis in Canada. Pathogens.

[29] Ferri M., Ranucci E., Romagnoli P., Giaccone V. (2017). Antimicrobial resistance: A global emerging threat to public health systems. Crit. Rev. Food Sci. Nutr..

[30] Eman M., Sharaf D.M. (2024). Comparing the effect of nitazoxanide and tylosin against necrotic enteritis in broilers. J. Adv. Vet. Res..

[31] Gomaa N.H., El-Aziz N.K.A., El-Naenaeey E.-s.Y., Abdelaziz W.S., Sewid A.H. (2023). Antimicrobial potential of myricetin-coated zinc oxide nanocomposite against drug-resistant *Clostridium perfringens*. BMC Microbiol..

[32] Immerseel F.V., Buck J.D., Pasmans F., Huyghebaert G., Haesebrouck F., Ducatelle R. (2004). *Clostridium perfringens* in poultry: An emerging threat for animal and public health. Avian Pathol..

[33] Kiu R., Hall L.J. (2018). An update on the human and animal enteric pathogen *Clostridium perfringens*. Emerg. Microbes Infect..

[34] Ibrahim A.H., El Khashab E., Shakal M., Morsy E.A. (2024). Unveiling antibiotic resistance, virulence, and molecular detection of enteric bacterial infections in broilers: A Study in Egypt. Egypt. J. Vet. Sci..

[35] Helal S.S., Khalaf N.M., El Menisy A.A., Lebdah M.A. (2019). *Clostridium perfringens* type A causing necrotic enteritis outbreaks among chickens in Egypt. Zagazig Vet. J..

[36] Zhang T., Zhang W., Ai D., Zhang R., Lu Q., Luo Q., Shao H. (2018). Prevalence and characterization of *Clostridium perfringens* in broiler chickens and retail chicken meat in central China. Anaerobe.

[37] Haider Z., Ali T., Ullah A., Basit A., Tahir H., Tariq H., Ilyas S.Z., Hayat Z., Rehman S.-u. (2022). Isolation, toxinotyping and antimicrobial susceptibility testing of *Clostridium perfringens* isolated from Pakistan poultry. Anaerobe.

[38] Rana E.A., Nizami T.A., Islam M.S., Barua H., Islam M.Z. (2023). Phenotypical identification and toxinotyping of *Clostridium perfringens* isolates from healthy and enteric disease-affected chickens. Vet. Med. Int..

[39] Yadav J.P., Kaur S., Dhaka P., Vijay D., Bedi J.S. (2022). Prevalence, molecular characterization, and antimicrobial resistance profile of *Clostridium perfringens* from India: A scoping review. Anaerobe.

[40] El-Gaos M., Khalil M., Abdelrahman M., Ramadan A. (2020). Molecular characterization of *Clostridium perfringens* isolated from turkeys. Assiut Vet. Med. J..

[41] Salem S.M., Mustafa D.I., Hamed R.I., El-Azzouny M.M., Anwar N. (2020). Assessment of pathological changes of mixed infection of coccidiosis and necrotic enteritis in turkey. J. Egypt. Vet. Med. Assoc..

[42] Yudiarti T., Sugiharto S., Widiastuti E., Wahyuni H., Sartono T., Nasution M. (2024). Physiological parameters, intestinal microbial population and internal organ weight of broilers supplemented with the fungus monascus purpureus. Adv. Anim. Vet. Sci..

[43] Soromou L.W., Leno P.F., Kamano A., Souare M.L., Camara A.O.D., Camara K. (2024). Current practices in the veterinary use of antibiotics in poultry laying hens in Friguiagbé (Guinea). J. Drug Deliv. Ther..

[44] Miyakawa M.E.F., Casanova N.A., Kogut M.H. (2024). How did antibiotic growth promoters increase growth and feed efficiency in poultry?. Poult. Sci..

[45] Schuetz A.N. (2014). Antimicrobial resistance and susceptibility testing of anaerobic bacteria. Clin. Infect. Dis..

[46] Gharieb R., Saad M., Abdallah K., Khedr M., Farag E., Abd El-Fattah A. (2021). Insights on toxin genotyping, virulence, antibiogram profiling, biofilm formation and efficacy of disinfectants on biofilms of *Clostridium perfringens* isolated from poultry, animals and humans. J. Appl. Microbiol..

[47] Slavić Đ., Boerlin P., Fabri M., Klotins K.C., Zoethout J.K., Weir P.E., Bateman D. (2011). Antimicrobial susceptibility of *Clostridium perfringens* isolates of bovine, chicken, porcine, and turkey origin from Ontario. Can. J. Vet. Res..

[48] Semenyuk E.G., Laning M.L., Foley J., Johnston P.F., Knight K.L., Gerding D.N., Driks A. (2014). Spore formation and toxin production in *Clostridium difficile* biofilms. PLoS ONE.

[49] Lu R., Liu B., Wu L., Bao H., García P., Wang Y., Zhou Y., Zhang H. (2023). A broad-spectrum phage endolysin (LysCP28) able to remove biofilms and inactivate *Clostridium perfringens* strains. Foods.

[50] Bai X., Nakatsu C.H., Bhunia A.K. (2021). Bacterial biofilms and their implications in pathogenesis and food safety. Foods.

[51] Jamal M., Ahmad W., Andleeb S., Jalil F., Imran M., Nawaz M.A., Hussain T., Ali M., Rafiq M., Kamil M.A. (2018). Bacterial biofilm and associated infections. J. Chin. Med. Assoc..

[52] Sheng Y., Chen Z., Wu W., Lu Y. (2023). Engineered organic nanoparticles to combat biofilms. Drug Discov. Today.

[53] Ahmed H.A., El Bayomi R.M., Hamed R.I., Mohsen R.A., El-Gohary F.A., Hefny A.A., Elkhawaga E., Tolba H.M. (2022). Genetic relatedness, antibiotic resistance, and effect of silver nanoparticle on biofilm formation by *Clostridium perfringens* isolated from chickens, pigeons, camels, and human consumers. Vet. Sci..

[54] Torky H.A., Khalil S.A., Elkassas F.A., Rezk M.S., Tawfik R.G. (2023). Effect of Silver nanoparticles on biofilm formation by *Clostridium perfringens* isolated from poultry and molecular typing of strains by ERIC-PCR. J. Adv. Vet. Res..

[55] Abdalkarim S.Y.H., Yu H.Y., Song M.L., Zhou Y., Yao J., Ni Q.Q. (2017). In vitro degradation and possible hydrolytic mechanism of PHBV nanocomposites by incorporating cellulose nanocrystal-ZnO nanohybrids. Carbohydr. Polym..

[56] Ikram M., Imran M., Hayat S., Shahzadi A., Haider A., Naz S., Ul-Hamid A., Nabgan W., Fazal I., Ali S. (2022). MoS_2_/cellulose-doped ZnO nanorods for catalytic, antibacterial and molecular docking studies. Nanoscale Adv..

[57] Worku L.A., Tadesse M.G., Bachheti R.K., Bachheti A., Husen A. (2024). Synthesis of carboxylated cellulose nanocrystal/ZnO nanohybrids using *Oxytenanthera abyssinica* cellulose and zinc nitrate hexahydrate for radical scavenging, photocatalytic, and antibacterial activities. Int. J. Biol. Macromol..

[58] Baldelli A., Etayash H., Oguzlu H., Mandal R., Jiang F., Hancock R.E., Pratap-Singh A. (2022). Antimicrobial properties of spray-dried cellulose nanocrystals and metal oxide-based nanoparticles-in-microspheres. Chem. Eng. J. Adv..

[59] Smith L.D., Holdeman L.V. (1968). The Pathogenic Anaerobic Bacteria.

[60] Quinn P., Markey B.K., Carter M., Donnelly W., Leonard F. (2002). Veterinary Microbiology and Microbial Disease.

[61] Yoo H.S., Lee S.U., Park K.Y., Park Y.H. (1997). Molecular typing and epidemiological survey of prevalence of *Clostridium perfringens* types by multiplex PCR. J. Clin. Microbiol..

[62] (2017). Performance Standards for Antimicrobial Susceptibility Testing: Twenty-Seventh Informational Supplement.

[63] Hu W.-S., Kim H., Koo O.K. (2018). Molecular genotyping, biofilm formation and antibiotic resistance of enterotoxigenic *Clostridium perfringens* isolated from meat supplied to school cafeterias in South Korea. Anaerobe.

[64] Andrews J. (2013). BSAC Methods for Antimicrobial Susceptibility Testing.

[65] Tambekar D., Dhanorkar D., Gulhane S., Khandelwal V., Dudhane M. (2006). Antibacterial susceptibility of some urinary tract pathogens to commonly used antibiotics. Afr. J. Biotechnol..

[66] Magiorakos A.-P., Srinivasan A., Carey R.B., Carmeli Y., Falagas M., Giske C.G., Harbarth S., Hindler J., Kahlmeter G., Olsson-Liljequist B. (2012). Multidrug-resistant, extensively drug-resistant and pandrug-resistant bacteria: An international expert proposal for interim standard definitions for acquired resistance. Clin. Microbiol. Infect..

[67] Freeman D., Falkiner F., Keane C. (1989). New method for detecting slime production by coagulase negative *Staphylococci*. J. Clin. Pathol..

[68] Tendolkar P.M., Baghdayan A.S., Gilmore M.S., Shankar N. (2004). Enterococcal surface protein, Esp, enhances biofilm formation by *Enterococcus faecalis*. Infect. Immun..

[69] O’Toole G.A., Kolter R. (1998). Initiation of biofilm formation in *Pseudomonas fluorescens WCS365* proceeds via multiple, convergent signalling pathways: A genetic analysis. Mol. Microbiol..

[70] Stepanović S., Vuković D., Hola V., Bonaventura G.D., Djukić S., Ćirković I., Ruzicka F. (2007). Quantification of biofilm in microtiter plates: Overview of testing conditions and practical recommendations for assessment of biofilm production by *Staphylococci*. Apmis.

[71] Choi O., Cho S.K., Kim J., Park C.G., Kim J. (2016). In vitro antibacterial activity and major bioactive components of *Cinnamomum verum* essential oils against cariogenic bacteria, *Streptococcus mutans* and *Streptococcus sobrinus*. Asian Pac. J. Trop. Biomed..

[72] Dalli M., Azizi S.-e., Benouda H., Azghar A., Tahri M., Bouammali B., Maleb A., Gseyra N. (2021). Molecular composition and antibacterial effect of five essential oils extracted from *Nigella sativa* L. seeds against multidrug-resistant bacteria: A Comparative study. Evid.-Based Complement. Altern. Med..

[73] Teh C.H., Nazni W.A., Nurulhusna A.H., Norazah A., Lee H.L. (2017). Determination of antibacterial activity and minimum inhibitory concentration of larval extract of fly via resazurin-based turbidometric assay. BMC Microbiol..

[74] Raja A.F., Ali F., Khan I.A., Shawl A.S., Arora D.S., Shah B.A., Taneja S.C. (2011). Antistaphylococcal and biofilm inhibitory activities of acetyl-11-keto-β-boswellic acid from Boswellia serrata. BMC Microbiol..

[75] Kalishwaralal K., BarathManiKanth S., Pandian S.R.K., Deepak V., Gurunathan S. (2010). Silver nanoparticles impede the biofilm formation by *Pseudomonas aeruginosa* and *Staphylococcus epidermidis*. Colloids Surf. B Biointerfaces.

[76] Papa R., Selan L., Parrilli E., Tilotta M., Sannino F., Feller G., Tutino M.L., Artini M. (2015). Anti-biofilm activities from marine cold adapted bacteria against *Staphylococci* and *Pseudomonas aeruginosa*. Front. Microbiol..

[77] Yasugi M., Okuzaki D., Kuwana R., Takamatsu H., Fujita M., Sarker M.R., Miyake M. (2016). Transcriptional profile during deoxycholate-induced sporulation in a *Clostridium perfringens* isolate causing foodborne illness. Appl. Environ. Microbiol..

[78] Soncini S.R., Hartman A.H., Gallagher T.M., Camper G.J., Jensen R.V., Melville S.B. (2020). Changes in the expression of genes encoding type IV pili-associated proteins are seen when *Clostridium perfringens* is grown in liquid or on surfaces. BMC Genom..

[79] Wu S.-B., Rodgers N., Choct M. (2011). Real-time PCR assay for *Clostridium perfringens* in broiler chickens in a challenge model of necrotic enteritis. Appl. Environ. Microbiol..

[80] Yuan J.S., Reed A., Chen F., Stewart C.N. (2006). Statistical analysis of real-time PCR data. BMC Bioinform..

[81] Team R.C. (2024). R: A Language and Environment for Statistical Computing.

[82] Wickham H., Chang W., Henry L., Pedersen T.L., Takahashi K., Wilke C., Woo K., Yutani H., Dunnington D., van den Brand T. (2007). ggplot2: Create Elegant Data Visualisations Using the Grammar of Graphics.

[83] Kolde R. (2010). Pheatmap: Pretty Heatmaps.

[84] Kassambara A., Mundt F. (2016). Factoextra: Extract and Visualize the Results of Multivariate Data Analyses.

