# Supplementary material for: Cellulose Nanocrystal/Zinc Oxide Bio-Nanocomposite Activity on Planktonic and Biofilm Producing Pan Drug-Resistant Clostridium perfringens Isolated from Chickens and Turkeys
Source: Antibiotics (Basel). 2025 Jun 3;14(6):575. doi: 10.3390/antibiotics14060575 (PMC12189287; doi:10.3390/antibiotics14060575)
Supplement: Supplementary file 1 [file antibiotics-14-00575-s001.zip › Table S2.pdf]

Table S2. Oligonucleotide primers used in the study.

Target gene	Function	Primers sequence 5 → 3`	Annealing temperature (° C)	References
16S rRNA	Species-specific for conventional PCR	F: AAAGATGGCATCATCATTCAAC<br>R: TACCGTCATTATCTTCCCCAAA	50	[61]
agrB	Quorum sensing	F: AAATATTCTGGAGGAGCACA<br>R: CATTGCCATGCTACTCCAAA	60	[77]
pilA2	Pili biosynthesis	F: TCAAATGCTAAAGACGACGTTACAG<br>R: TCTACTGCAAATACACCACCATCAA		[78]
16S rRNA	Housekeeping gene	F: CGCATAACGTTGAAAGATGG<br>R: CCTTGGTAGGCCGTTACCC		[79]
