# Supplementary figures and images for: Cellulose Nanocrystal/Zinc Oxide Bio-Nanocomposite Activity on Planktonic and Biofilm Producing Pan Drug-Resistant Clostridium perfringens Isolated from Chickens and Turkeys
Source: Antibiotics (Basel). 2025 Jun 3;14(6):575. doi: 10.3390/antibiotics14060575 (PMC12189287; doi:10.3390/antibiotics14060575)

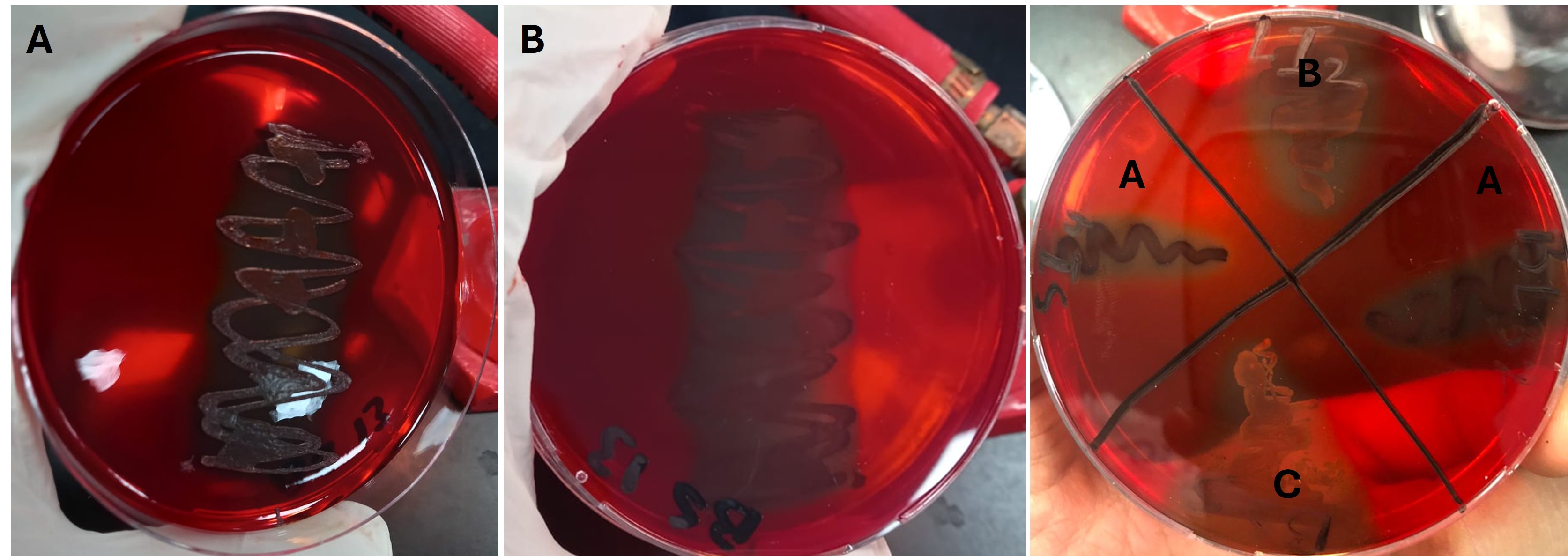

Supplement: Supplementary file 1 [file antibiotics-14-00575-s001.zip › Figure S1.jpg]

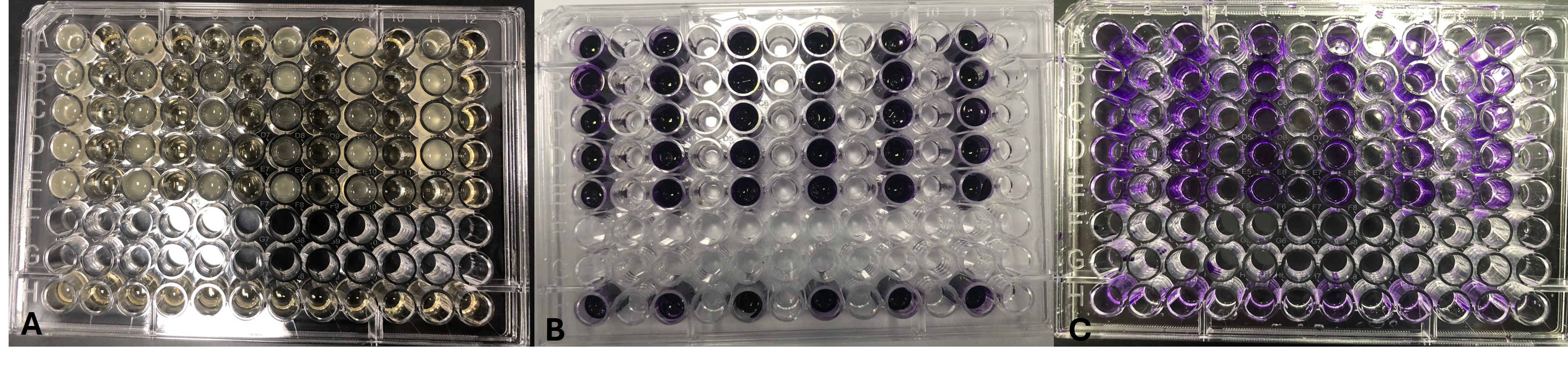

Supplement: Supplementary file 1 [file antibiotics-14-00575-s001.zip › Figure S2.jpg]

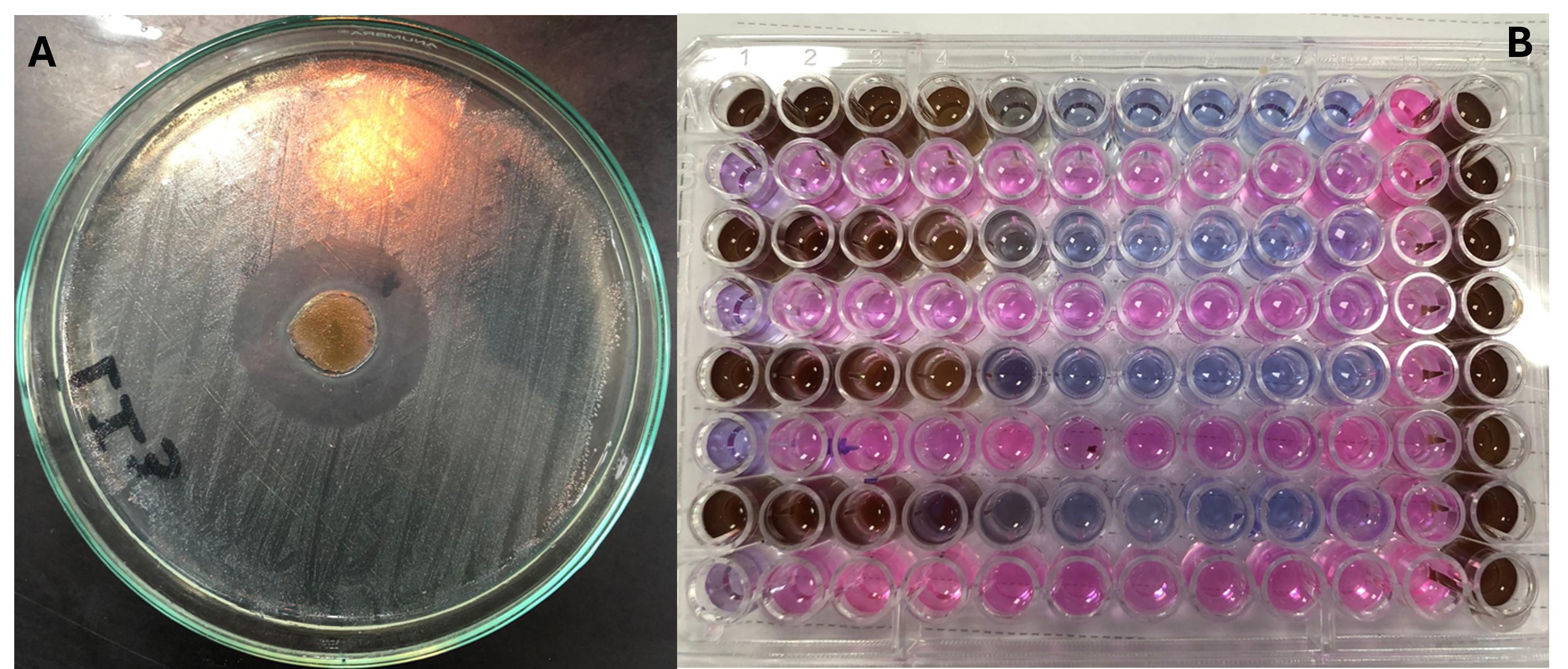

Supplement: Supplementary file 1 [file antibiotics-14-00575-s001.zip › Figure S3.jpg]

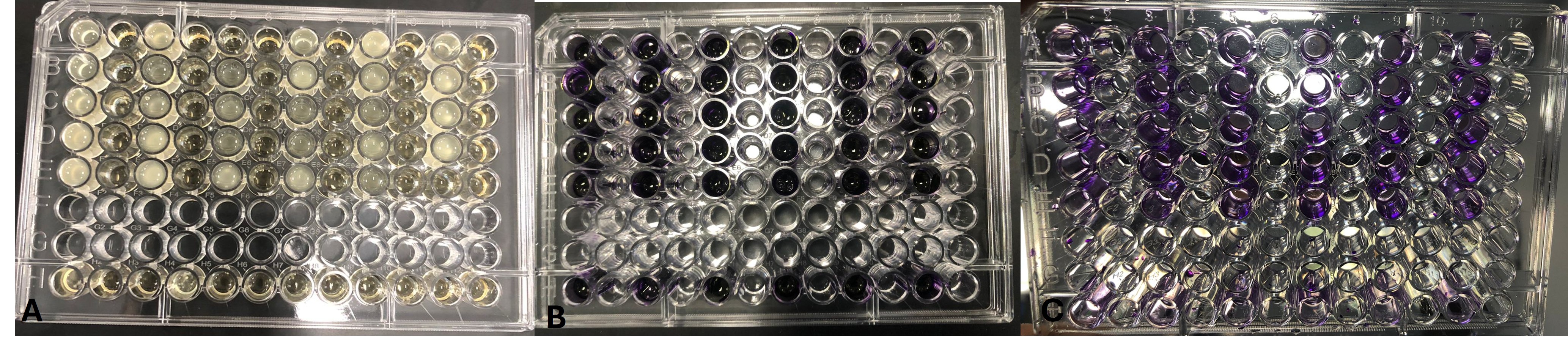

Supplement: Supplementary file 1 [file antibiotics-14-00575-s001.zip › Figure S4.jpg]
